# Exploring the Allosteric Pathways of Asciminib in the Dual Inhibition of BCR-ABL1

**DOI:** 10.3390/biom15091214

**Published:** 2025-08-22

**Authors:** Jie Ming, Hongwei Gao, Jiuyu Zhan

**Affiliations:** School of Life Science, Ludong University, Yantai 264025, China; 20192504430@m.ldu.edu.cn (J.M.); zhanjy@ldu.edu.cn (J.Z.)

**Keywords:** BCR-ABL1, CML, tyrosine kinase inhibitors, allosteric inhibition, combination therapy

## Abstract

The BCR-ABL1 fusion protein is a critical therapeutic target in Chronic Myeloid Leukemia (CML). Current monotherapy approaches involve types of inhibitors that can be categorized into ATP competitive inhibitors and allosteric inhibitors. However, resistance mutations in the tyrosine kinase domain of BCR-ABL1 have limited the effectiveness of these drugs. Research indicates that dual inhibition of BCR-ABL1 by combining these two types of inhibitors effectively addresses the issue of drug resistance as there are no overlapping resistance mechanisms. However, the underlying reasons for the observed synergistic effects have not yet been thoroughly elucidated. In this study, we employed molecular dynamics simulation to observe the synergistic interactions of BCR-ABL1 by the allosteric inhibitor asciminib and ATP competitive inhibitors nilotinib and ponatinib. Our study reveals that when asciminib binds to BCR-ABL1, nilotinib and ponatinib exhibit more substantial binding stability compared to monotherapy. At the atomic level, we have elucidated the reasons for the enhanced binding affinity of nilotinib and ponatinib when using a co-inhibition therapy. Our study reveals the allosteric communication pathway between asciminib and ponatinib, providing more detailed insights into the effectiveness of combination therapy. These findings provide valuable insights into combination therapies, aiding in the rational use of medications and guiding the design of novel inhibitors.

## 1. Introduction

Chronic myeloid leukemia (CML) is a clonal myeloproliferative neoplasm that accounts for approximately 15% of newly diagnosed leukemia cases in adults [1]. It is characterized by the presence of the Philadelphia chromosome, a genetic abnormality resulting from a reciprocal translocation between chromosomes 9 and 22, which leads to the fusion of the Abelson tyrosine kinase (ABL1) gene with the breakpoint cluster region (BCR) gene, encoding the constitutively active tyrosine kinase (BCR-ABL1) and driving the uncontrolled proliferation of myeloid cells [2,3]. In the treatment of CML there has been a concentrated effort to target the BCR-ABL1 fusion protein, which serves as a key therapeutic target [3,4]. The initial therapeutic approach centered on the use of inhibitors designed to obstruct ATP binding to the tyrosine kinase active site specifically, consequently suppressing the activity of BCR-ABL1 [5].

In 2001, imatinib was approved by the FDA for front-line treatment of CML, marking a significant milestone as the first-generation BCR-ABL1 inhibitor [6]. The success of imatinib validated the feasibility of targeted therapy for CML through kinase inhibitors. However, the emergence of resistance due to point mutations with the kinase domain has hindered imatinib’s ability to completely cure CML [7].

**Figure 1:**
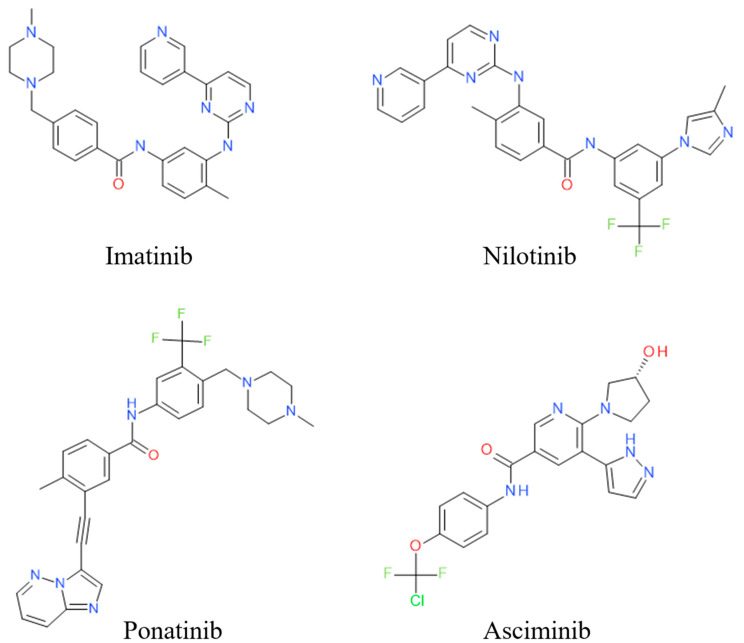
Two-dimensional structure of c-ABL inhibitors.

**Figure 2:**
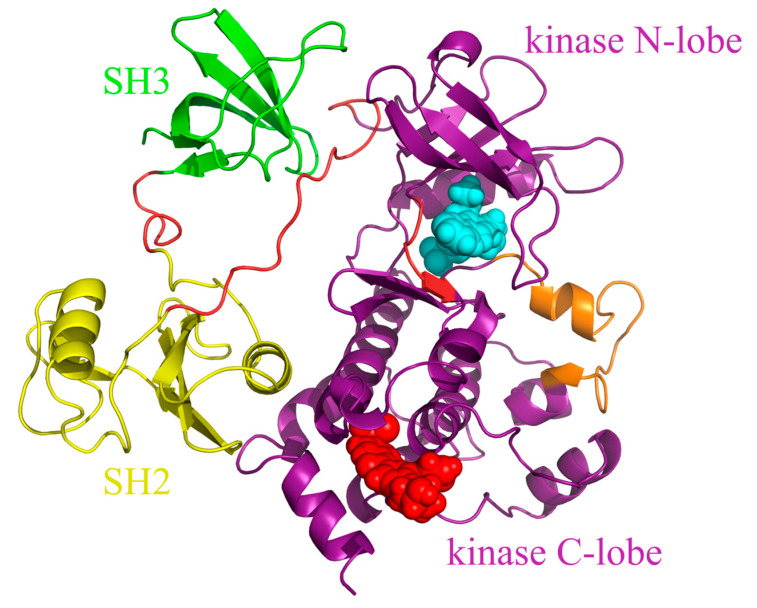
The three-dimensional structure of the ABL1 protein (PDB ID code 5MO4). The inhibitor nilotinib (cyan) is bound to the ATP site, whereas asciminib (red) is bound to the allosteric site in the kinase domain. Color coding is as follows: SH3 domain (green), SH2 domain (yellow), kinase domain (deep purple), interdomain linkers (tv-red), and activation loop (orange).

To address this problem, the second-generation tyrosine kinase inhibitors (TKIs) nilotinib and dasatinib were developed using distinct design approaches [8,9]. Nilotinib, based on the structure of imatinib, underwent structural optimization to enhance its interactions with residues at the ATP site of inactive ABL, thereby increasing its binding affinity to ABL1 [10,11,12]. Like imatinib, nilotinib is classified as a “DFG-out” inhibitor [10]. Dasatinib, on the other hand, targets a conserved region distant from the ATP site to achieve binding with the active conformation of ABL1, making it a “DFG-in” inhibitor [13,14]. Although second-generation inhibitors possess higher potency than imatinib and are effective against most imatinib-resistant mutants, they are ineffective against the “gatekeeper” T334I mutation (T315I in Abl 1a numbering) [15,16,17,18]. The third-generation inhibitors such as ponatinib have been developed to target the T334I mutation, binding at the ATP site of the inactive conformation of ABL1 as a “DFG-out” inhibitor [11]. However, ponatinib has been associated with severe adverse reactions, and resistance can emerge due to the emergence of T334M mutation or compound mutations, rendering ponatinib ineffective [19,20,21,22]. The fourth-generation inhibitor asciminib (Figure 1) was approved by the FAD in 2021 for the clinical treatment of CML [23,24]. Asciminib mimics the myristoyl group, binding to the myristoyl pocket (allosteric site), thereby restoring the ABL1 protein’s autoinhibitory mechanism and shifting the protein conformation to an inactive state, which inhibits the activity of BCR-ABL1 [23,25,26]. The allosteric site is distinct from the ATP site, which lays the foundation for the combination therapy of ATP competitive inhibitors with allosteric inhibitors (Figure 2) [27].

Resistance and drug toxicity have limited the application of TKIs in the clinical treatment of CML. However, the combination therapy of ATP competitive inhibitors and allosteric inhibitors holds promise in overcoming these issues [28]. Jahnke et al. provides evidence that asciminib can co-bind with ATP site inhibitors, including imatinib, nilotinib, and dasatinib, to the ABL protein. Importantly, this co-binding does not compromise the individual inhibitory mechanisms of these agents [29]. The study indicates the feasibility of combining allosteric inhibitors with ATP competitive inhibitors for the treatment of ABL protein. Combination therapy with drugs that lack overlapping resistance mechanisms can effectively reduce the emergence of drug resistance [28,30]. In 2024, Okamoto et al. showed that the combined use of the ATP competitive inhibitor imatinib and the allosteric asciminib prevented the development of resistant mutations [31]. Another potential advantage of combination therapy is that it leverages synergistic effects between drugs, effectively reducing the required dosages. Wylie et al. and Gleixner et al. indicate that the co-administration of asciminib with nilotinib or ponatinib enhances the inhibition against BCR-ABL1 and suppresses the emergence of resistance mutations [32,33,34]. The addition of asciminib significantly reduces the concentration of ponatinib required to achieve inhibition [34]. As the field of combination therapy advances, it is essential to gain a deeper understanding of the structural basis for the synergistic effects of drug molecules under dual inhibition to further develop TKIs with enhanced efficacy [33,35].

In this study, we aim to explore the inhibitory mechanism of allosteric inhibitor asciminib in conjunction with ATP competitive inhibitors to induce conformational inactivation of ABL1. Through molecular dynamics simulations, we investigate how the binding of asciminib affects the inhibitory activity of ATP competitive inhibitors nilotinib and ponatinib against wild-type (WT) ABL1 at the molecular level. In this study, we aim to decipher the inhibitor-residue interactions under co-inhibition, enhancing our understanding of the communication between the ATP binding site and the allosteric site of ABL1.

## 2. Materials and Methods

### 2.1. System Preparation

For this study, five different systems were considered: ABL1–asciminib, ABL1–nilotinib, ABL1–nilotinib–asciminib, ABL1–ponatinib, and ABL1–ponatinib–asciminib. The initial structure of the ABL1 protein was retrieved from Protein Data Bank (PDB code:5MO4), which corresponds to the ABL1 kinase domain with the T334I and D382N mutations in complex with asciminib and nilotinib [32]. Two reverse mutations were carried out, and the missing residues were restored using Discovery Studio 2020 (DS 2020) to obtain the complete WT ABL1 structure. Subsequently, DS 2020 was utilized for molecular docking to position ponatinib into the ATP binding site of the WT ABL1 protein. The ionizable residues within the ABL1 structure were assigned their protonation states using the H++ server [36].

### 2.2. Molecular Dynamics (MD) Simulations

MD simulations were carried out on the systems with AMBER 18 [37]. The partial charges and missing parameter fields for the inhibitors were determined using the Antechamber module in AMBER 18 software. The inhibitors were parameterized with the General AMBER Force Field (GAFF) [38], while ABL1 was parameterized with the ff14SB force [39]. The t-leap module was employed to ensure the electrical neutrality of the system, with the protein complex positioned at the center of the TIP3P water model [40].

All of the MD simulations were executed by the AMBER 18 software [41]. A two-stage minimization process was employed to prepare a reasonable initial model. Initially, the complex was minimized under constraints (500 kcal mol^−1^ Ǻ^−2^) for 10,000 steps. Subsequently, the minimization was repeated for another 10,000 steps without any restrictions. The system was gradually heated from 0 K to 310 K over a period of 300 ps, followed by an equilibration simulation at a constant temperature of 310 K for 1ns. Under NPT conditions, we conducted 200 ns MD simulations for four complex systems to obtain trajectories. Subsequently, the trajectories were analyzed using the cpptraj module.

### 2.3. MM-GB/SA Calculations

Determining the binding free energy for protein–ligand interactions is a significant approach within the field of computational biophysics. To calculate the free binding energy between inhibitors and the ABL1 protein, we employed the MM-GBSA (Molecular Mechanics Generalized Born Surface Area) method in AMBER 18 [42,43,44,45]. The binding free energy (${\Delta G}_{\mathrm{bind}}$) was derived from an average of 10,000 snapshots extracted from each simulation trajectory ranging from 100 ns to 200 ns. The calculation is based on the following formula:

(1) $$\Delta G_{\mathrm{bind}}=G_{\mathrm{complex}}-{(G}_{\mathrm{receptor}}+G_{\mathrm{ligand}})$$

(2) $$\Delta G_{\mathrm{bind}}=\Delta E_{\mathrm{MM}}+\Delta G_{\mathrm{sol}}-T\Delta S$$

(3) $$\Delta E_{\mathrm{MM}}=\Delta E_{\mathrm{int}}+\Delta E_{\mathrm{ele}}+\Delta E_{\mathrm{vdW}}$$

(4) $${\Delta G}_{\mathrm{sol}}{=\Delta G}_{\mathrm{GB}}+\Delta G_{\mathrm{SA}}$$

(5) $$\Delta G_{\mathrm{SA}}=\gamma SASA$$

where the terms $G_{\mathrm{complex}}$, $G_{\mathrm{receptor}}$, and $G_{\mathrm{ligand}}$ symbolize the free energy of the complex, the receptor, and the ligand. The terms $\Delta E_{\mathrm{MM}}$, $\Delta G_{\mathrm{sol}}$, and TΔS symbolize the gas-phase energy, solvation free energy, and a vibrational entropy term. $\Delta E_{\mathrm{MM}}$ is divided into internal interaction ($\Delta E_{\mathrm{int}}$), electrostatic interaction ($\Delta E_{\mathrm{ele}}$), and van der Waals energy ($\Delta E_{\mathrm{vdW}}$). Solvation free energy (${\Delta G}_{\mathrm{sol}}$) is the summation of polar solvation energy (${\Delta G}_{\mathrm{GB}}$) and nonpolar solvation energy ($\Delta G_{\mathrm{SA}}$). The ${\Delta G}_{\mathrm{GB}}$ contribution was assessed by solving the GB equation, and the $\Delta G_{\mathrm{SA}}$ was determined from the solvent accessible surface area (SASA). We performed free energy calculations utilizing the Generalized Born (GB) model with the igb = 5 parameter set. For the calculation of the nonpolar solvation free energy term, γ is set to 0.0072 kcal mol^−1^ Ǻ^−2^ and SASA is the solvent accessible surface area determined by a probe radius of 1.4 Ǻ.

When analyzing a ternary complex, we treat the inhibitor of interest as the ligand and consider the protein bound to the second small molecule as the receptor. Thus, to compute the binding free energy of nilotinib in the ABL1–nilotinib–asciminib system, nilotinib is designated as the ligand, while the ABL1–asciminib complex is regarded as the receptor.

To further elucidate the interactions between the protein and inhibitors, we dissected the total binding free energy to the residue level using the MM-GB/SA method in AMBER 18, based on the stable trajectory obtained from molecular dynamics simulations.

### 2.4. Residue Interaction Network

In this study, the webPSN web server was employed to analyze the residue interaction networks of the systems, enhancing our comprehension of allosteric communication within biomolecular systems [46]. Based on the final 100 ns of the MD trajectories, PSNTools was utilized to generate networks detailing the interactions between protein residues [47]. In our study, a residue network was constructed by treating each amino acid residue and ligand as a node, with non-covalent interactions between residues serving as edges to connect these nodes. The webPSN server was also used to compute differences in residue networks between single- and dual-inhibition systems, thereby identifying allosteric communication paths caused by asciminib binding to the ABL1 protein.

## 3. Results and Discussion

### 3.1. Correlation of ATP Binding Site with Allosteric Site

**Figure 3:**
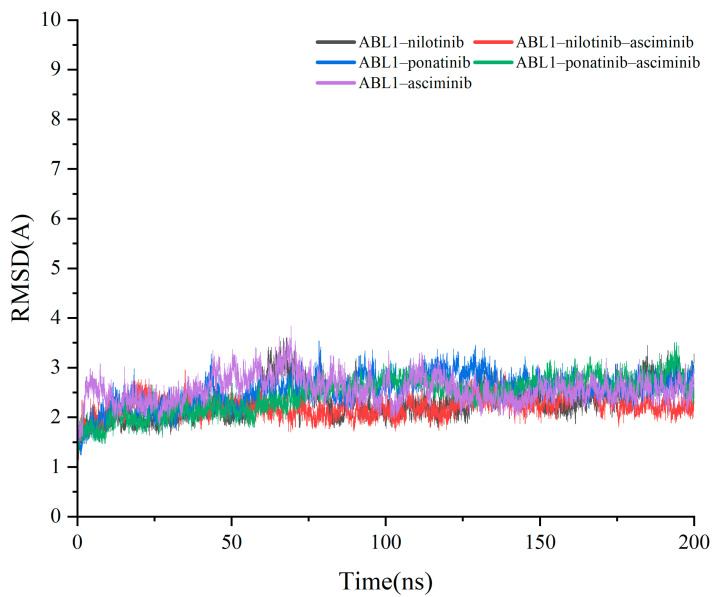
RMSD of Cα atoms of the protein backbone atoms.

The stability of the complex system was assessed by measuring the root mean square deviation (RMSD) of the complex structure. The average RMSD for all C-α atoms of the trajectories for all the systems demonstrate that equilibration was achieved after 100 ns and can be used for the following analyses (Figure 3).

**Figure 4:**
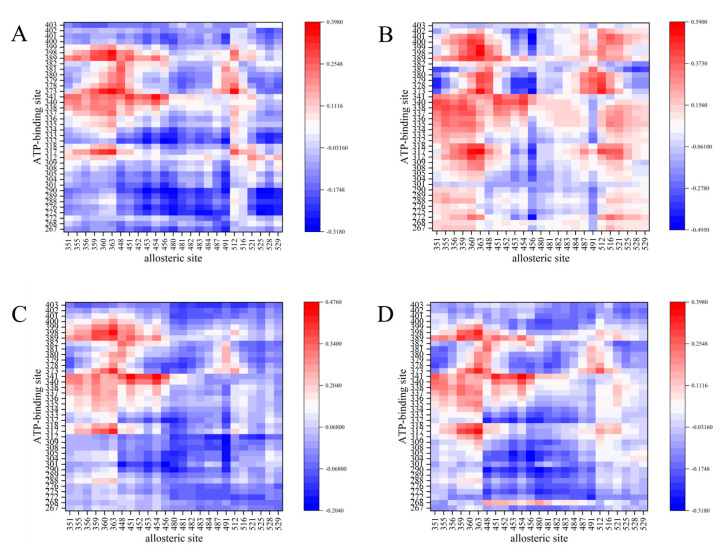
Dynamic cross correlation of ATP binding site and allosteric site of the BCR-ABL1. (**A**) ABL1–nilotinib, (**B**) ABL1–nilotinib–asciminib, (**C**) ABL1–ponatinib, (**D**) ABL1–ponatinib–asciminib. The color relates to the degree of correlation, the higher the correlation, the darker the color. Red is a highly positive correlation; blue is a highly negative correlation.

Dynamic cross correlation (DCC) analyses are a popular method for analyzing communications among separate parts of a molecular system [48,49]. The method can decipher the hidden dynamics of residues in the ATP binding site and allosteric site of BCR-ABL1. To investigate the effect of double-drugging for BCR-ABL1, we focus on the positive correlation of atoms within 5 Ǻ from inhibitors (Figure 4). An occurrence of positive concerted motion between residues indicates the presence of strong interactions among them, which means coordinated movement in the same direction. As shown in Figure 4, the results show a stabilizing pathway between the ATP binding site and the allosteric site of BCR-ABL1, suggesting that the co-binding of asciminib and nilotinib enhances the communication of ATP binding site and allosteric site of BCR-ABL1. When asciminib binds to ABL1 protein, it promotes cooperative movements between nilotinib and a larger number of residues at the ATP binding site. This suggests that more residues are engaged in allosteric communication. However, the dynamic correlation coefficient (DCC) only indicates the dynamic coupling between the ATP site and the allosteric site. To further analyze how asciminib binding to ABL1 affects the binding affinity of ATP inhibitors at the ATP site, a more in-depth analysis through the calculation of binding free energy is necessary.

### 3.2. Binding Free Energy Calculations

To evaluate the binding energy of the ATP competitive inhibitors and allosteric inhibitors when they bind to BCR-ABL1 alone, as well as during co-inhibition, the MM-GB/SA method in AMBER18 was used in calculating the four systems and is shown in Table 1. The binding free energy (${\Delta G}_{\mathrm{bind}}$) indicates that the value of binding free energy of nilotinib or ponatinib to ABL1 was higher when bound alone. The results demonstrate that the co-inhibition of BCR-ABL1 by nilotinib or ponatinib and asciminib results in a more favorable binding than when each inhibitor is bound alone.

From the comparison between the single-inhibition system and the co-inhibition system, it was found that the enhancement in the binding affinity of ponatinib with ABL1 under co-inhibition is primarily attributed to the strengthened electrostatic interactions between ponatinib and the residues of the ATP binding site.

In the co-inhibition system, the binding affinity of asciminib to ABL1 is slightly reduced compared with that in the ABL1–asciminib complex alone, a result that aligns with experimental observations (Table 2). When asciminib is co-bound with an ATP competitive inhibitor to the ABL1 protein, a weak antagonism is observed; however, this does not prevent the simultaneous engagement of both inhibitors at ABL1, and their combination still yields synergistic therapeutic efficacy [29].

In order to further provide a detailed understanding of each residue that contributes significantly to the binding of ATP competitive inhibitors to BCR-ABL1, both when they bind alone and during co-inhibition with asciminib, we decomposed the total binding free energy into the binding free energies of individual residues. The energy of key residues, which includes van der Waals energy, electrostatic interaction, polar solvation energy, and nonpolar solvation energy, is characterized by nonpolar solvation and van der Waals interaction energies as positive contributors to the binding of inhibitors. In contrast, polar solvation energy acts as a negative factor affecting it. The key residues with binding free energy (${\Delta G}_{\mathrm{bind}}\leq \;-1\;\mathrm{kcal}/\mathrm{mol}$) are listed in Tables S1–S4. Residues showing a free energy change of 20% or more are considered to have a significant impact on the allosteric communication pathway (Table 3). The co-binding of nilotinib with ABL1 and asciminib results in decreased binding affinity for key residues PHE378, ASH400, and PHE401. Interestingly, when asciminib binds to ABL1 it diminishes the binding affinity of nilotinib for key residues that are crucial in allosteric communication, as seen in the ABL1–nilotinib–asciminib system compared to the ABL1–nilotinib system alone. In other words, the enhanced binding affinity of nilotinib for ABL1 in the presence of asciminib is mainly attributed to non-key residues rather than key residues. In light of the DCC analysis, the binding of asciminib to ABL1 protein has been shown to engage a larger number of residues at the ATP binding site in cooperative movements with residues at the allosteric site. When compared to the nilotinib–ABL1 system, this suggests that interactions between a greater number of residues and nilotinib have been enhanced. However, there is no significant qualitative improvement observed. The increased binding affinity between nilotinib and ABL1 protein represents a quantitative advancement rather than a qualitative one. In contrast, co-binding of ponatinib with ABL1 and asciminib enhances the binding affinity for key residues LEU267, VAL275, THR334, and GLU335, but weakens it for the key residue PHE336.The improved binding affinity of ponatinib with residues LEU267, VAL275, and THR334 is attributed to enhanced van der Waals interaction energies, indicating a closer approach of these residues to ponatinib. In contrast, the increased binding affinity between GLU335 and ponatinib stems from strengthened electrostatic interactions. The result indicates that the presence of asciminib enhances the binding affinity between key residues and ponatinib, thereby strengthening the overall binding affinity of ponatinib to ABL1.

### 3.3. The H-Bonding Ability of Inhibitors at the ATP Binding Site

**Figure 5:**
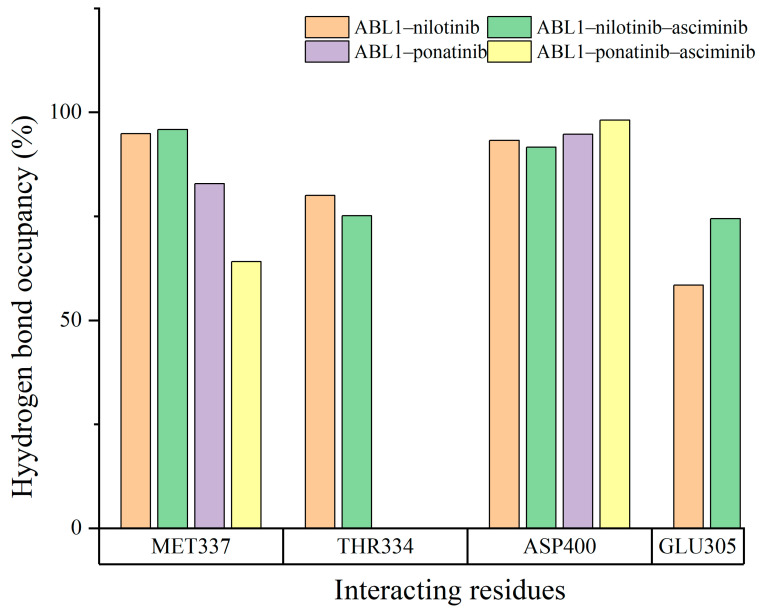
The hydrogen bond occupancies for key residues at the ABL1 binding interface with ATP competitive inhibitors. Only residues with hydrogen bond occupancies exceeding 40% are depicted.

To evaluate the impact of the binding of the allosteric inhibitor asciminib on the H-bonding ability of ATP competitive inhibitors nilotinib and ponatinib at the ATP site, hydrogen bond occupancies were used for four systems, and the results are shown in Figure 5. In the ABL1–nilotinib system, the hydrogen bond occupancy value for GLU305 in their interaction with nilotinib was 58.44%. In the ABL1–nilotinib–asciminib system, the value was 74.45% for GLU305. The hydrogen bond interaction between nilotinib and GLU305 at the ATP site was enhanced when asciminib was also bound. In the ABL1–ponatinib system, the hydrogen bond occupancy value for MET337 in the interaction with ponatinib was 82.84%. In the ABL1–ponatinib–asciminib system, the value changed to 64.14%. The results revealed that the presence of asciminib impeded the formation of hydrogen bonds between ponatinib and MET337. From the hydrogen bond occupancy results, it can be inferred that the binding of asciminib to ABL1 affects the hydrogen occupancies between inhibitors and residues at the ATP site, thereby influencing the binding affinity between inhibitors and individual residues. However, when considering the inhibitors as a whole, these changes in hydrogen bond occupancies do not significantly impact the binding affinity between the inhibitors and the ABL1 protein.

Further analysis from the perspective of the entire inhibitor molecule was conducted to observe the allosteric inhibitor asciminib on the hydrogen bonding ability of ATP competitive inhibitors at the ATP site. The average number of hydrogen bonds formed by ATP competitive inhibitors in the systems was calculated as follows: nilotinib in ABL1–nilotinib (3.32), nilotinib in ABL1–nilotinib–asciminib (3.40), ponatinib in ABL1–ponatinib (2.03), ponatinib in ABL1–ponatinib–asciminib (1.92). From the perspective of the inhibitors’ overall hydrogen bonding ability, the changes in hydrogen bonding ability between the inhibitors and the ABL1 protein, induced by asciminib binding to ABL1, do not significantly affect the binding affinity of ATP competitive inhibitors to ABL1.

### 3.4. The Hydrophobic Interaction of Inhibitors at the ATP Binding Site

**Figure 6:**
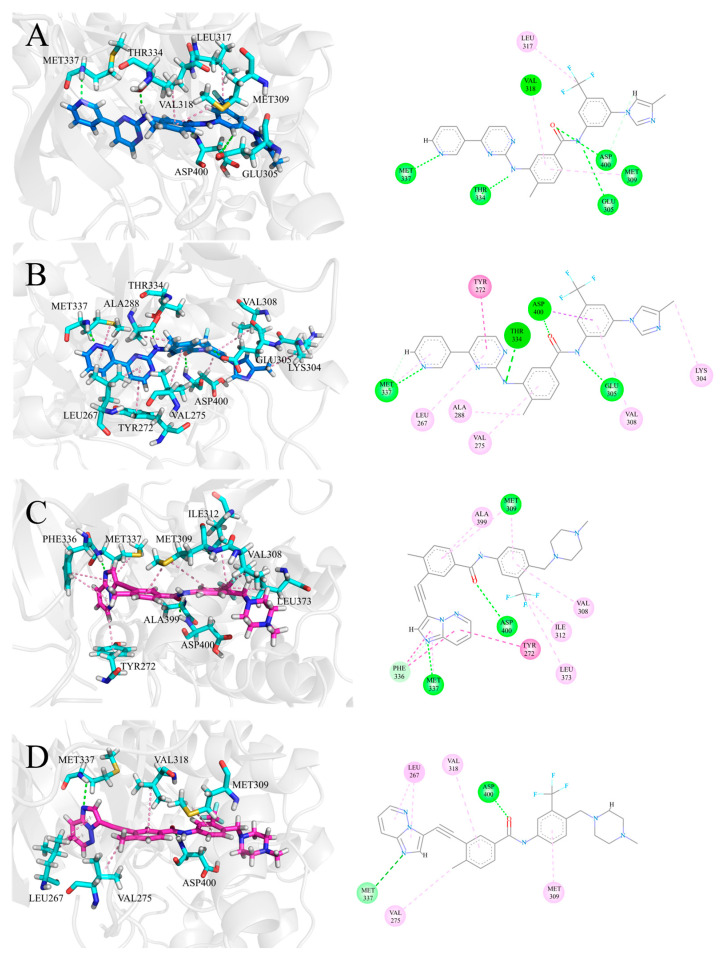
Binding modes of inhibitors to ABL1 are illustrated, with nilotinib and ponatinib shown in marine and light magenta. Residues with interactive relationships are displayed in stick mode, and H-bonds and hydrophobic interactions are marked with green and pink dotted lines. Nitrogen atoms are marked in blue, sulfur atoms in yellow, and oxygen atoms in red. The left side of the figure shows the three-dimensional structures, and the right side shows the two-dimensional representations. In the right-side figure: Inhibitors are shown as lines, residues as balls, hydrogen bonds as green dashed lines, hydrophobic interactions as pink dashed lines, and residues are colored by interaction type. (**A**) Nilotinib binding to residues in the ABL1–nilotinib system. (**B**) Nilotinib binding to residues in the ABL1–nilotinib–asciminib system. (**C**) Ponatinib binding to residues in the ABL1–ponatinib system. (**D**) Ponatinib binding to residues in the ABL1–ponatinib–asciminib system.

To further analyze the impact of the interaction between ATP competitive inhibitors and residues at the ATP site when co-inhibited with allosteric inhibitor asciminib in ABL1, we focused on the hydrophobic interactions formed by inhibitors and residues, which are a significant factor in evaluating the binding of ATP competitive inhibitors to the ATP site of ABL1, and the results are shown in Figure 6. In the ABL1–nilotinib–asciminib system, nilotinib forms hydrophobic interactions with residues ASP400, TYR272, ALA288, LYS304, VAL308, VAL275, LEU267, and MET337 at the ATP site, leading to a more stable binding of nilotinib in the ATP site. Additionally, the hydrophobic interactions between nilotinib and LEU267, TYR272, VAL275, and ASP400 resulted in a displacement of nilotinib position compared to the ABL1–nilotinib system. In the ABL1–ponatinib–asciminib system, the hydrophobic interactions formed between ponatinib and residues LEU267, VAL275, MET309, and VAL318 at the ATP site enhanced the binding stability of ponatinib with ABL1. Based on the analysis of binding free energies of the residues, it was found that LEU267 and VAL318 play a crucial role in the binding of ponatinib to ABL1 in the ABL1–ponatinib–asciminib system. The results suggest that these hydrophobic interactions are crucial for maintaining the binding affinity of inhibitors, as they help stabilize the interaction between inhibitors and residues in the ATP site.

### 3.5. The Electrostatic Interaction of Inhibitors at the ATP Binding Site

**Figure 7:**
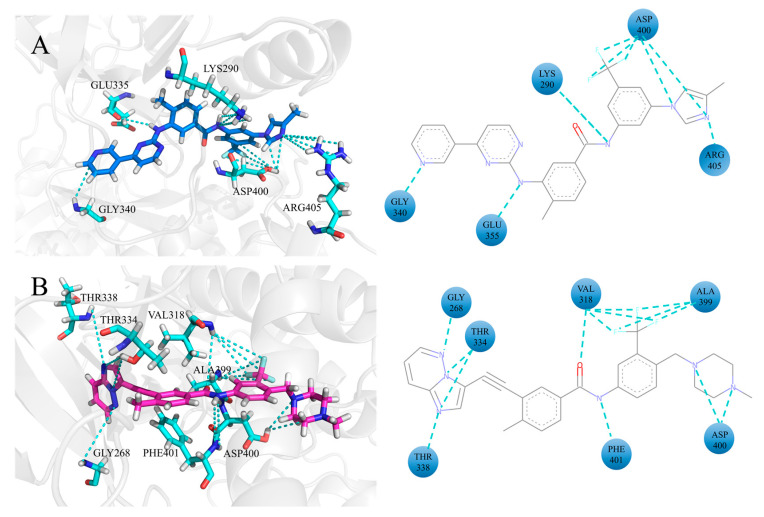
Electrostatic interactions between nilotinib and ponatinib with the ATP binding site residues are depicted. Nilotinib is shown in marine blue, while ponatinib is represented in light magenta. Residues with interactive relationships are displayed in stick mode, and electrostatic interactions are marked with cyan dotted lines. Nitrogen atoms are marked in blue and oxygen atoms in red. The left panel presents the three-dimensional view, whereas the right panel provides a two-dimensional representation. In the right-side figure, inhibitors are shown as lines, residues are shown as blue balls, and electrostatic interactions are shown as cyan dashed lines. (**A**) Nilotinib binding to residues in the ABL1–nilotinib–asciminib system. (**B**) Ponatinib binding to residues in the ABL1–ponatinib–asciminib system.

The enhancement of electrostatic interactions between ATP competitive inhibitors (nilotinib and ponatinib) and ABL1 is a significant factor contributing to the increased binding affinity of these inhibitors with ABL1 when they co-inhibit ABL1 with asciminib. To further elucidate the impact of these interactions, this study focused on the electrostatic interactions between ATP competitive inhibitors and each residue at the ATP site during the co-inhibition of ABL1 by ATP competitive inhibitors and asciminib. Summarizing the electrostatic interactions between ATP competitive inhibitors and ABL1 with an occupancy rate greater than 40% in both the single-inhibition system and the co-inhibition system, we identified residues that demonstrate enhanced interactions with ATP competitive inhibitors compared to the single-inhibition system, as shown in Figure 7.

In the ABL1–nilotinib–asciminib system, the residues that exhibit enhanced electrostatic interactions with nilotinib are rather sparsely distributed. This scattered distribution pattern suggests that the electrostatic contributions to binding are not localized to a single region but rather spread across various parts of the protein, which have implications for the overall binding affinity and specificity of the complex. In the ABL1–ponatinib–asciminib system, residues VAL318, ALA399, ASP400, and PHE401 encircled ponatinib, forming stronger electrostatic with the retroamide group and the trifluoromethyl group. The retroamide group is known to enhance the potency of ponatinib and anchor the inhibitor in the ATP site [11]. In contrast, the trifluoromethyl group contributes to exploring new hydrophobic contacts in the deep region of the ATP site [11]. The results suggest that the enhanced electrostatic interactions between the retroamide group and residues play a crucial role in increasing the affinity of ponatinib for ABL1 during the co-inhibition of ABL1 by ponatinib and asciminib.

In this study, we employed molecular docking and molecular dynamics simulations to establish four systems that model the inhibition of ABL tyrosine kinase by ATP competitive inhibitors—nilotinib and ponatinib—either alone or in combination with asciminib within the human body. Through dynamic cross correlation (DCC) analyses, we determined that the ATP site and allosteric site are not entirely independent. Instead, they exhibit positive concerted motion, indicating the presence of a stable signaling pathway between the two. This suggests that the binding of asciminib to ABL1 can influence the binding of ATP competitive inhibitors to ABL1 through signal transduction mechanisms.

Nilotinib, developed through the structural optimization of imatinib, retains the same hydrogen bonding capabilities as imatinib while favorably expanding hydrophobic interactions at the ATP site, thereby enhancing the therapeutic effect against imatinib-resistant mutations [12]. Interestingly, when co-bound with asciminib to ABL1, nilotinib and imatinib exhibit distinctly different binding affinities with ABL1 [30]. The binding free energy of key residues decreases, indicating a weakened binding capacity between nilotinib and these residues. This suggests that the presence of asciminib affects the binding of nilotinib with key residues at the ATP site, leading to a reduction in their binding affinity. The enhancement of nilotinib’s binding affinity to ABL1 in the presence of asciminib is indeed an intriguing phenomenon. This increase is not attributed to key residues but rather to the improvement in binding free energy of non-key residues, which is a noteworthy observation. Similarly, the presence of asciminib has a minimal impact on the overall hydrogen bonding ability of nilotinib but significantly affects its hydrophobic interactions. Nilotinib forms hydrophobic interactions with a more significant number of residues at the ATP site, thereby enhancing its binding affinity to ABL1. In DCC analyses, when nilotinib co-inhibits ABL1 with asciminib there is a significant positive concerted motion between more residues at the ATP site and residues at the allosteric site compared to when nilotinib inhibits ABL1 alone.

Synthesizing our analysis, we venture a hypothesis: given that asciminib binds to the allosteric site of ABL1, resulting in reduced binding energy between nilotinib and key residues at the ATP site, we would have expected asciminib to hinder the binding of nilotinib to the ATP site of ABL1. However, the structural advantages of nilotinib allow it to maintain its binding affinity with ABL1. The residues at the ATP site do not effectively “push” nilotinib out; instead, they enhance interactions with nilotinib, forming additional hydrophobic contacts and strengthening nilotinib’s hydrophobic capabilities at the ATP site. This enhancement is sufficient to explain the increased binding affinity between nilotinib and ABL1, which relies on the binding free energy of non-key residues. The strengthened hydrophobic interactions between the ATP site residues and nilotinib bind nilotinib with more residues, leading to concerted motion. This also accounts for the observed increase in residues exhibiting positive concerted motion at the ATP site under co-inhibition.

Ponatinib is based on AP23464, a trisubstituted purine analog, and has undergone structural optimization to enhance its drug efficacy [11]. Based on the results, it is evident that the binding of asciminib to ABL1 enhances the binding affinity of ponatinib with ABL1, with the improvement in binding affinity of ponatinib relying on strengthening interactions with key residues. The combination of asciminib with ABL1 intensifies the electrostatic interactions between partial groups of ponatinib and residues at the ATP site. The combined therapy of asciminib and ponatinib in the treatment of ABL1 initiates communication between residues at the allosteric site and those at the ATP site. The binding of asciminib to ABL1 enhances the strong interactions between partial groups of ponatinib and key residues through signal transduction, thereby influencing the binding of ponatinib to ABL1.

### 3.6. Allosteric Communications Between Asciminib and Ponatinib

Based on the ABL1–ponatinib system and the ABL1–ponatinib–asciminib system, we used PSNTools to calculate the interactions between residues over the time range of 100 ns to 200 ns, exploring the potential communication mechanisms between asciminib and ponatinib.

To explore the allosteric communication pathways by which asciminib binding to ABL1 enhances ponatinib’s binding affinity to ABL1, we screened the global metapath using asciminib and ponatinib as start and end points, creating new metapaths. The relevant information of the filtered paths is summarized in Table 4. The increased number of nodes and links in the metapath of the ABL1–ponatinib–asciminib system, compared to the ABL1–ponatinib system, indicate that asciminib binding to ABL1 can broaden communication paths between residue pairs and activate allosteric communication pathways. The decrease in the number of shortest paths indicates that the binding of asciminib to ABL1 optimizes the pathways for signal transmission, thereby enhancing the efficiency of signal propagation. The average path length, which refers to the average number of nodes in the filtered paths pool, indicates that a higher value suggests that signal transmission in the residue network involves more amino acids, highlighting the intricate nature of ABL1′s allosteric communication function. The changes in these values indicate that the ABL1 protein exhibits greater stability and efficiency under the combined inhibition of ponatinib and asciminib.

**Figure 8:**
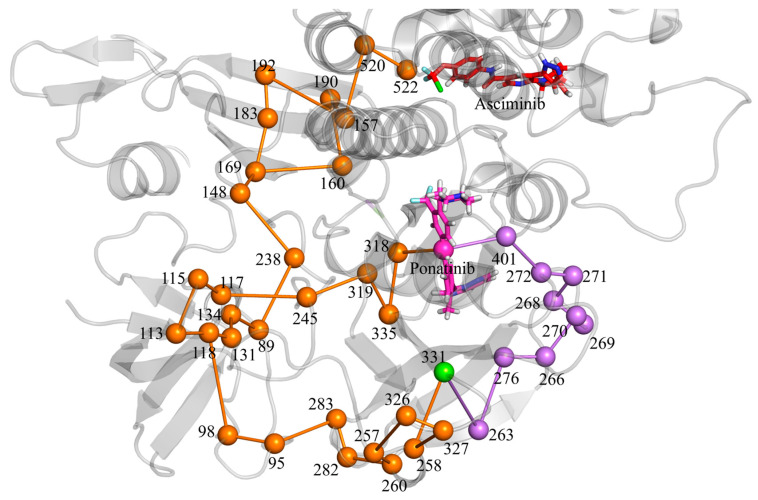
Three-dimensional diagram of the communication pathway between asciminib and ponatinib. Nodes and links specific to the ABL1–ponatinib–asciminib system network are colored orange, those specific to the ABL1–ponatinib system network are colored purple, and nodes and links shared by both networks are colored green. Asciminib is shown as a red stick, ponatinib is displayed as a light magenta stick and node, where nitrogen atoms are blue, chlorine atoms are green, and fluorine atoms are cyan.

Our study focuses on the allosteric communication pathway between the allosteric site and the ATP binding site (Figure 8). This pathway is initiated by residue SER522 at the allosteric site, traverses the SH2 domain, and upon reaching residue TRP118 in the SH3 domain diverges into two distinct signaling routes. One signaling pathway passes through residues ASN113 → ASN115 → GLU117 → TYR245 → GLN319 → GLU335 → VAL318 → ponatinib. Another pathway passes through residues THR98 → GLY95 → TYR283 → LYS282 → ASP260 → GLU257 → ARG326 → GLU327 → ARG258 → TYR331 → MET263 → TYR276 → LYS266 → GLY270 → GLY269 → GLY268 → GLN271 → TYR272 → PHE401 → ponatinib.

**Figure 9:**
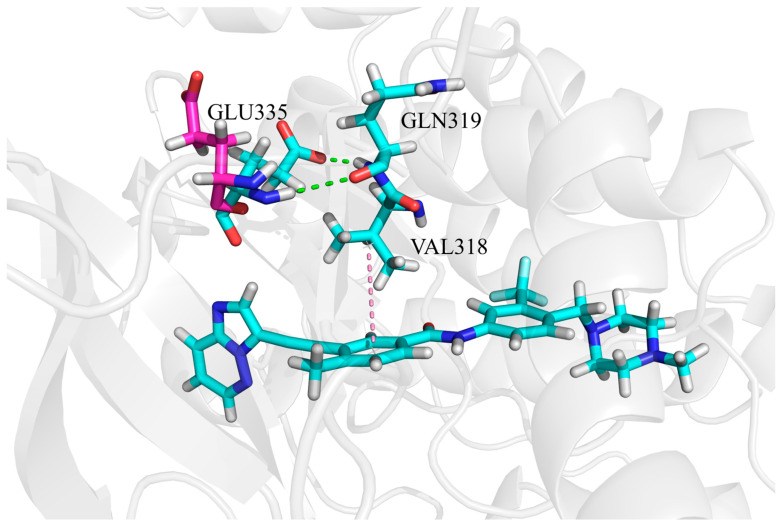
Interactions of residues GLU335, GLN319, and VAL318 with ponatinib. Residues in the ABL1–ponatinib system are shown in light magenta, while residues in the ABL1–ponatinib–asciminib system are shown in cyan. Residues are displayed in stick mode. H-bonds and hydrophobic interactions are marked with green and pink dotted lines, respectively. Nitrogen atoms are displayed in blue, and oxygen atoms are in red.

The first pathway is associated with the enhanced binding affinity between GLU335 and ponatinib (Figure 9). Specifically, the carboxyl group of GLU335 forms a hydrogen bond with the nitrogen atom of GLN319. This interaction draws the negatively charged carboxyl group of GLU335 closer to ponatinib, thereby facilitating electrostatic interactions. This explains why electrostatic interactions play a crucial role in the enhanced binding affinity between GLU335 and ponatinib.

**Figure 10:**
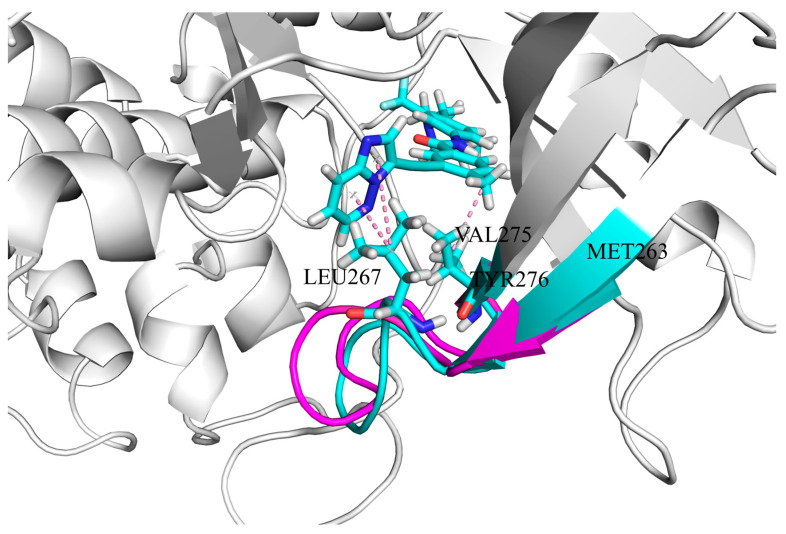
Interactions of ponatinib with residues LEU267 and VAL275. Residues in the ABL1–ponatinib system (MET263 → TYR276) are shown in light magenta, while residues in the ABL1–ponatinib–asciminib system (MET263 → TYR276) are shown in cyan. Residues LEU267 and VAL275 are displayed in stick mode, and hydrophobic interactions are marked with pink dotted lines. Nitrogen atoms are displayed in blue, and oxygen atoms are in red.

The second pathway is associated with enhanced binding affinity between residues LEU267, VAL275, and ponatinib (Figure 10). The TRP118 → TYR331 segment is unique to the ABL1–ponatinib–asciminib system, while TYR331 → PHE401 is specific to the ABL1-ponatinib system. This indicates that asciminib binding to ABL1 disrupts the TYR331 → PHE401 signaling pathway. The allosteric communication in the ABL1–ponatinib–asciminib system causes the protein conformation MET263 → TYR276 to be more open than in the ABL1–ponatinib system. This allows residues LEU267 and VAL275 to move closer to the hydrophobic groups of ponatinib, strengthening the hydrophobic interactions between them.

## 4. Conclusions

Mutations in the tyrosine kinase domain of the BCR-ABL1 fusion protein pose a significant challenge to the treatment of chronic myeloid leukemia (CML). Combination therapy is emerging as a promising new strategy in addressing this issue. The combination therapy of asciminib with ATP competitive inhibitors has been shown to effectively control and eliminate CML xenograft tumors, with no recurrence observed after treatment cessation. Interestingly, in the combined treatment of asciminib with nilotinib or ponatinib, the required doses of nilotinib or ponatinib were significantly reduced [50]. This suggests that we can achieve therapeutic effects with lower drug doses, thereby reducing the potential side effects and toxicity associated with the drugs. This study employed molecular dynamics (MD) simulation and molecular mechanics generalized born surface area (MM-GB/SA) to investigate the interactions between inhibitors and residues at the ATP site when asciminib binds to ABL1. This study elucidates the molecular mechanisms underlying the impact of asciminib binding to ABL1 on the binding affinity of ATP competitive inhibitors, including nilotinib and ponatinib, with ABL1. The study also clarifies that the difference in binding affinity between imatinib and nilotinib with ABL1, when asciminib is bound to ABL1, is attributed to the inherent binding affinity of the inhibitors themselves with ABL1. The successful combination therapy of asciminib with ponatinib has effectively addressed the issue of ponatinib’s limited use due to its severe side effects. This study indicates that the enhancement of ponatinib’s binding affinity to ABL1 relies on the strengthened interactions between certain groups of ponatinib and residues at the ATP site. The research findings contribute to the potential for further structural optimization of ponatinib, with the expectation of developing inhibitors with lower toxic side effects when used in combination with asciminib for the treatment of ABL1.

The study provides a molecular understanding of the combined treatment of ABL1, and our results can aid in the further design and development of these inhibitors to enhance their inhibitory effects in the treatment of CML. This understanding is crucial for optimizing therapeutic strategies and potentially leads to the discovery of inhibitors with lower toxic side effects, which is particularly important in the context of combination therapies involving asciminib and other ATP competitive inhibitors like nilotinib and ponatinib.

## Figures and Tables

**Table 1:** Binding energy of four complex at the ABL1 ATP binding site (${\mathrm{kcal}\;\mathrm{mol}}^{-1}$).

System	${\Delta E}_{\mathrm{vdw}}$	${\Delta E}_{\mathrm{ele}}$	${\Delta G}_{\mathrm{GB}}$	${\Delta G}_{\mathrm{SA}}$	−TΔS	${{\Delta G}_{\mathrm{bind}}}^{\;a}$
ABL1–nilotinib	−71.75	−34.5284	45.075	−9.1536	31.5174	−38.8396
ABL1–nilotinib–asciminib	−72.3503	−37.3234	47.6184	−9.4476	30.2918	−41.2111
ABL1–ponatinib	−72.3503	−23.5641	29.3745	−9.0356	29.3277	−44.5496
ABL1–ponatinib–asciminib	−73.4241	−30.6382	34.2425	−9.3242	27.285	−51.859

***^a^***: ${\Delta G}_{bind}={\Delta G}_{MM-GB/SA}-T\Delta S.$$\;{\Delta G}_{MM-GB/SA}={\Delta E}_{vdw}+{\Delta E}_{ele}+{\Delta G}_{GB}+{\Delta G}_{SA}.$

**Table 2:** Binding energy of three complex at the ABL1 allosteric site (${\mathrm{kcal}\;\mathrm{mol}}^{-1}$).

System	${\Delta E}_{vdw}$	${\Delta E}_{ele}$	${\Delta G}_{GB}$	${\Delta G}_{SA}$	−TΔS	${{\Delta G}_{bind}}^{\;a}$
ABL1–ascminib	−43.7724	−24.8216	33.4048	−5.7043	22.6313	−18.2622
ABL1–nilotinib–asciminib	−43.9577	−22.5531	35.7391	−5.6030	22.0603	−14.3145
ABL1–ponatinib–asciminib	−44.9838	−19.3029	33.4972	−5.6402	21.3559	−15.0738

***^a^***: ${\Delta G}_{bind}={\Delta G}_{MM-GB/SA}-T\Delta S.$$\;{\Delta G}_{MM-GB/SA}={\Delta E}_{vdw}+{\Delta E}_{ele}+{\Delta G}_{GB}+{\Delta G}_{SA}.$

**Table 3:** Summary of binding free energy on key residues affected by allosteric modulation (${\mathrm{kcal mol}}^{-1}$).

Inhibitor	Residues	${{\Delta G}_{\mathrm{bind}}}^{1}$	${{\Delta G}_{\mathrm{bind}}}^{2}$	Percentage Change
nilotinib	PHE378	−1.014	−0.695	−31%
	ASP400	−2.865	−2.305	−20%
	PHE401	−1.926	−1.55	−20%
ponatinib	LEU267	−0.844	−1.575	87%
	VAL275	−0.847	−1.573	86%
	THR334	−1.256	−1.76	40%
	GLU335	−0.574	−1.014	77%
	PHE336	−1.669	−0.87	−48%

${{\Delta G}_{\mathrm{bind}}}^{1}$: the binding free energy of the residues within the ABL1–nilotinib system or the ABL1–ponatinib system. ${{\Delta G}_{\mathrm{bind}}}^{2}$: the binding free energy of the residues within the ABL1–nilotinib–asciminib system or ABL1–ponatinib–asciminib system.

**Table 4:** Summary of filtered paths differences between the ABL1–ponatinib system and the ABL1–ponatinib–asciminib system.

**Value**	**ABL1–Ponatinib–Asciminib System**	**ABL1–Ponatinib System**
Number Of Nodes in MetaPath	73	45
Specific Nodes in MetaPath	48 (65.75%)	20 (44.44%)
Shared Nodes in MetaPath	25 (34.25%)	25 (55.56%)
Number Of Links MetaPath	73	45
Specific Links in MetaPath	53 (72.60%)	25 (55.56%)
Shared Links in MetaPath	20 (27.40%)	20 (44.44%)
Number of Shortest Paths	2039	2359
Length Of Smallest Path	3	4
Average Path Length	18.78	16.16
Length of Longest Path	38	31
Minimum Path Force	2.75	3.80
Average Path Force	7.34	8.17
Maximum Path Force	11.17	13.62
Minimum Path Correlation	0.80	0.80
Average Path Correlation	0.89	0.88
Maximum Path Correlation	0.94	0.93
Minimum % Of Corr. Nodes	2.78	3.45
Average % Of Corr. Nodes	9.27	10.51
Maximum % Of Corr. Nodes	100	75
Minimum Path Hubs %	10	0
Average Path Hubs %	42.66	38.64
Maximum Path Hubs %	80	75

## Data Availability

Data and software used in this work are all openly available. The initial structure of the ABL1 protein (PDB code:5MO4) was obtained from the RCSB Protein Data Bank (https://www.rcsb.org) (accessed on 11 March 2024). The structures of nilotinib, ponatinib, and asciminib were obtained from PubChem (https://pubchem.ncbi.nlm.nih.gov) (accessed on 13 March 2024). All of software utilized in this work, Discovery Studio 2020, H++ server, Amber18, and WebPSN are available since we have the license for each one.

## Supplementary Materials

The following supporting information can be downloaded at: https://www.mdpi.com/article/10.3390/biom15091214/s1, Figure S1: Dynamic Cross-Correlation of Residues in the Allosteric Communication Path; Table S1: Binding free energy decomposition on individual residues in the ABL1- nilotinib system (${\mathrm{kcal}\;\mathrm{mol}}^{-1}$); Table S2: Binding free energy decomposition on individual residues in the ABL1-nilotinib-asciminib system (${\mathrm{kcal}\;\mathrm{mol}}^{-1}$); Table S3: Binding free energy decomposition on individual residues in the ABL1-ponatinib system (${\mathrm{kcal}\;\mathrm{mol}}^{-1}$); Table S4: Binding free energy decomposition on individual residues in the ABL1-ponatinib-asciminib system (${\mathrm{kcal}\;\mathrm{mol}}^{-1}$).
