# Supplementary material for: Exploring the Allosteric Pathways of Asciminib in the Dual Inhibition of BCR-ABL1
Source: Biomolecules. 2025 Aug 22;15(9):1214. doi: 10.3390/biom15091214 (PMC12467782; doi:10.3390/biom15091214)
Supplement: Supplementary file 1 [file biomolecules-15-01214-s001.zip › biomolecules-3777046-supplementary.pdf]

Table S1. Binding free energy decomposition on individual residues in the ABL1- nilotinib system ($kcal\ mol^{-1}$).

	ΔE_{vdw}	ΔE_{ele}	ΔG_{GB}	ΔG_{SA}	ΔG_{bind}
LEU267	-1.2	0.085	0.056	-0.135	-1.194
TYR272	-1.536	-0.313	-0.116	-0.074	-2.039
VAL275	-1.209	-0.053	-0.04	-0.124	-1.426
ALA288	-1.384	-0.326	0.452	-0.101	-1.359
LYS290	-1.339	2.213	-1.405	-0.124	-0.655
GLU305	-1.99	-8	10.054	-0.257	-0.194
VAL308	-1.235	0.267	0.005	-0.164	-1.127
MET309	-2.12	-0.795	0.228	-0.176	-2.863
ILE312	-0.514	-0.125	0.187	-0.07	-0.522
LEU317	-0.655	-0.487	0.278	-0.048	-0.911
VAL318	-1.88	-0.23	0.59	-0.114	-1.634
ILE332	-0.847	-0.119	0.097	-0.028	-0.896
THR334	-1.618	-1.979	0.145	-0.139	-3.591
GLU335	-0.365	0.674	-0.231	-0.011	0.067
PHE336	-1.348	-1.345	0.5	-0.064	-2.258
MET337	-0.537	-2.178	1.613	-0.053	-1.156
LEU373	-0.344	-0.13	0.14	-0.035	-0.369
PHE378	-0.991	-0.077	0.262	-0.208	-1.014
LEU389	-1.72	0.006	-0.066	-0.181	-1.962
ALA399	-1.375	-1.097	0.932	-0.052	-1.592
ASH400	-3.003	-2.219	2.711	-0.354	-2.865
PHE401	-1.726	-0.185	0.139	-0.154	-1.926
GLY402	-0.084	0	0.126	0	0.042
LEU403	-0.026	-0.054	0.085	0	0.005

Table S2. Binding free energy decomposition on individual residues in the ABL1-nilotinib-asciminib system ($kcal\ mol^{-1}$).

	ΔE_{vdw}	ΔE_{ele}	ΔG_{GB}	ΔG_{SA}	ΔG_{bind}
LEU267	-1.211	0.113	0.023	-0.15	-1.225
TYR272	-1.484	-0.342	0.083	-0.076	-1.82
VAL275	-1.327	-0.039	-0.064	-0.129	-1.559
ALA288	-1.337	-0.259	0.452	-0.087	-1.231
LYS290	-1.401	2.365	-1.675	-0.121	-0.832
GLU305	-2.016	-8.568	10.344	-0.243	-0.483
VAL308	-1.446	0.349	-0.069	-0.197	-1.363
MET309	-2.041	-0.095	-0.11	-0.166	-2.411
ILE312	-0.665	-0.119	0.154	-0.07	-0.701
LEU317	-0.549	-0.393	0.201	-0.045	-0.785
VAL318	-1.346	-0.193	0.041	-0.09	-1.588
ILE332	-0.938	-0.149	0.047	-0.028	-1.068
THR334	-1.573	-1.902	-0.078	-0.138	-3.691
GLU335	-0.467	0.666	-0.421	-0.011	-0.234
PHE336	-1.278	-1.273	0.533	-0.049	-2.067
MET337	-0.702	-2.253	1.738	-0.076	-1.293
LEU373	-0.505	-0.144	0.145	-0.059	-0.563
PHE378	-0.736	-0.081	0.225	-0.104	-0.695
LEU389	-1.591	0.044	-0.098	-0.171	-1.817
ALA399	-1.242	-1.423	0.903	-0.07	-1.832
ASH400	-2.996	-2.197	3.226	-0.338	-2.305
PHE401	-1.379	-0.337	0.291	-0.124	-1.55
GLY402	-0.15	-0.101	0.235	-0.013	-0.03
LEU403	-0.051	-0.009	0.078	0	0.018

Table S3. Binding free energy decomposition on individual residues in the ABL1-ponatinib system ($kcal\ mol^{-1}$).

	ΔE_{vdw}	ΔE_{ele}	ΔG_{GB}	ΔG_{SA}	ΔG_{bind}
LEU267	-0.986	0.196	0.085	-0.139	-0.844
TYR272	-0.617	-0.241	0.22	-0.054	-0.692
VAL275	-0.797	-0.06	0.053	-0.042	-0.847
ALA288	-1.467	-0.36	0.548	-0.118	-1.397
LYS290	-1.232	2.309	-1.732	-0.09	-0.745
GLU305	-1.239	-5.712	8.388	-0.179	1.257
VAL308	-1.207	0.286	-0.594	-0.235	-1.751
MET309	-2.361	-0.896	0.08	-0.208	-3.385
ILE312	-0.98	-0.178	0.047	-0.124	-1.235
LEU317	-0.56	-0.513	0.145	-0.043	-0.971
VAL318	-1.726	-0.048	0.3	-0.114	-1.588
ILE332	-0.806	-0.143	0.031	-0.041	-0.959
THR334	-1.284	-0.068	0.207	-0.11	-1.256
GLU335	-0.36	-1.025	0.821	-0.011	-0.574
PHE336	-1.499	-0.653	0.532	-0.049	-1.669
MET337	-0.977	-1.346	1.258	-0.041	-1.106
LEU373	-0.715	-0.223	0.091	-0.089	-0.935
PHE378	-1.715	-0.372	0.362	-0.196	-1.92
LEU389	-1.765	-0.07	0.004	-0.183	-2.014
ALA399	-1.184	-1.593	0.766	-0.053	-2.064
ASH400	-2.52	-2.044	2.415	-0.384	-2.533
PHE401	-1.926	-0.072	0.449	-0.183	-1.733
GLY402	-0.051	-0.093	0.083	0	-0.062
LEU403	-0.016	-0.023	0.047	0	0.008

Table S4. Binding free energy decomposition on individual residues in the ABL1-ponatinib-asciminib system ($kcal\ mol^{-1}$).

	ΔE_{vdw}	ΔE_{ele}	ΔG_{GB}	ΔG_{SA}	ΔG_{bind}
LEU267	-1.596	-0.454	0.695	-0.221	-1.575
TYR272	-0.551	-0.298	0.317	-0.059	-0.59
VAL275	-1.326	-0.137	-0.013	-0.096	-1.573
ALA288	-1.342	-0.254	0.574	-0.079	-1.101
LYS290	-1.23	2.526	-1.91	-0.114	-0.727
GLU305	-1.082	-6.211	8.393	-0.129	0.972
VAL308	-1.309	0.512	-0.531	-0.259	-1.587
MET309	-2.293	-0.947	0.146	-0.223	-3.317
ILE312	-1.047	-0.209	0.05	-0.128	-1.334
LEU317	-0.73	-0.677	0.262	-0.076	-1.221
VAL318	-1.626	-0.105	0.058	-0.123	-1.797
ILE332	-0.758	-0.117	-0.025	-0.019	-0.919
THR334	-1.812	0.176	0.01	-0.134	-1.76
GLU335	-0.287	-0.627	-0.087	-0.013	-1.014
PHE336	-1.074	-0.294	0.514	-0.016	-0.87
MET337	-1.097	-1.142	1.232	-0.053	-1.06
LEU373	-0.82	-0.346	0.185	-0.092	-1.073
PHE378	-1.453	-0.379	0.391	-0.167	-1.608
LEU389	-1.76	-0.051	-0.047	-0.172	-2.029
ALA399	-1.2	-1.605	0.928	-0.054	-1.931
ASH400	-2.74	-4.719	5.711	-0.408	-2.156
PHE401	-1.907	-0.241	0.72	-0.146	-1.574

GLY402	-0.161	0.295	-0.309	-0.003	-0.178
LEU403	-0.661	0.197	-0.108	-0.195	-0.767

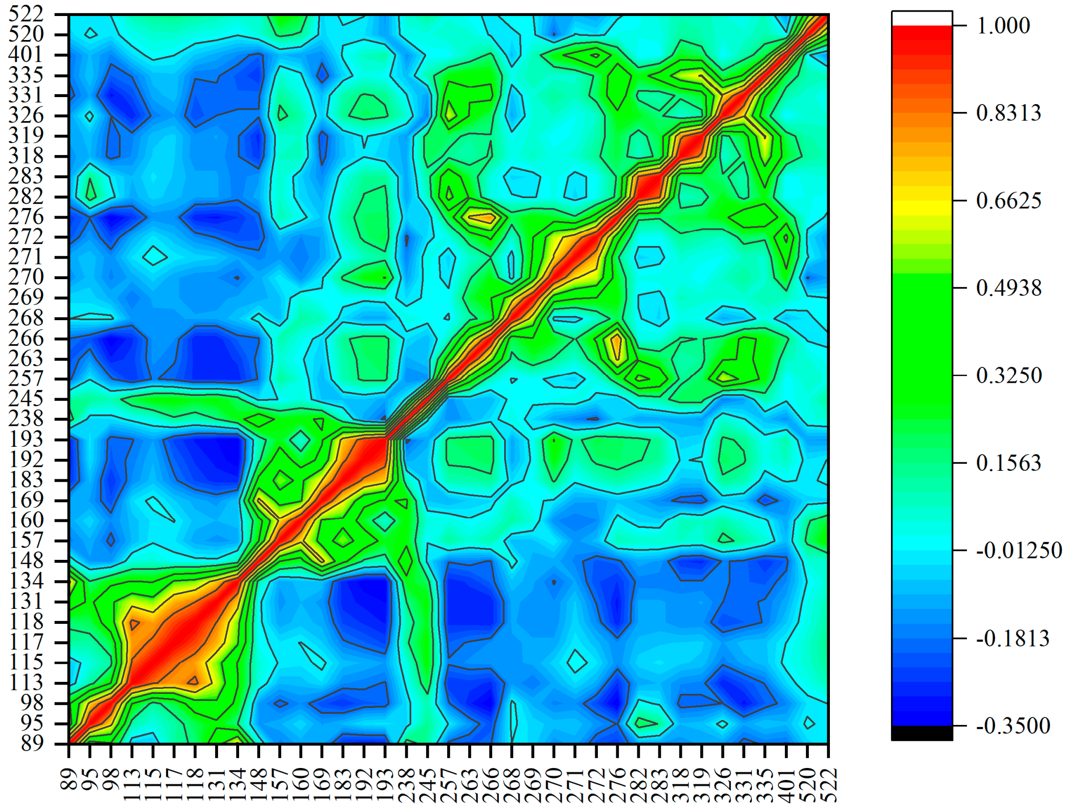

Figure S1. Dynamic Cross-Correlation of Residues in the Allosteric Communication Path
